# Gerbil: a fast and memory-efficient *k*-mer counter with GPU-support

**DOI:** 10.1186/s13015-017-0097-9

**Published:** 2017-03-31

**Authors:** Marius Erbert, Steffen Rechner, Matthias Müller-Hannemann

**Affiliations:** Institute of Computer Science, Martin Luther University Halle-Wittenberg, Von-Seckendorff-Platz 1, 06120 Halle (Saae), Germany

**Keywords:** *k*-mer counting, de novo assembly, Genome sequences, GPU computing, Algorithm engineering

## Abstract

**Background:**

A basic task in bioinformatics is the counting of *k*-mers in genome sequences. Existing *k*-mer counting tools are most often optimized for small *k* < 32 and suffer from excessive memory resource consumption or degrading performance for large *k*. However, given the technology trend towards long reads of next-generation sequencers, support for large *k* becomes increasingly important.

**Results:**

We present the open source *k*-mer counting software *Gerbil* that has been designed for the efficient counting of *k*-mers for *k* ≥ 32. Our software is the result of an intensive process of algorithm engineering. It implements a two-step approach. In the first step, genome reads are loaded from disk and redistributed to temporary files. In a second step, the *k*-mers of each temporary file are counted via a hash table approach. In addition to its basic functionality, *Gerbil* can optionally use GPUs to accelerate the counting step. In a set of experiments with real-world genome data sets, we show that *Gerbil* is able to efficiently support both small and large *k*.

**Conclusions:**

While *Gerbil*’s performance is comparable to existing state-of-the-art open source *k*-mer counting tools for small *k* < 32, it vastly outperforms its competitors for large *k*, thereby enabling new applications which require large values of *k*.

**Electronic supplementary material:**

The online version of this article (doi:10.1186/s13015-017-0097-9) contains supplementary material, which is available to authorized users.

## Background

The counting of *k*-mers in genome reads is a common task in bioinformatics. The problem is to count the occurrences of all *k*-long substrings in a large amount of sequencing reads. Its most prominent application is de novo assembly of genome sequences. Although building a histogram of *k*-mers seems to be quite a simple task from an algorithmic point of view, it has attracted a considerable amount of attention in recent years. In fact, the counting of *k*-mers in large genome sequences becomes a challenging problem, if it has to be both resource- and time-efficient and therefore makes it an interesting object of study for algorithm engineering. Existing tools for *k*-mer counting are often optimized for *k* < 32 and lack good performance for larger *k*. However, recent advances in technology towards larger read lengths are leading to the quest to cope with values of *k* exceeding 32. Studies elaborating on the optimal choice for the value of *k* recommend relatively high values for various applications [1, 2]. In particular, working with long sequencing reads helps to improve accuracy and contig assembly (with *k* values in the hundreds) [3]. In this paper, we introduce a tool with a high performance for such large values of *k*. A preliminary version of this article has been published in the proceedings of WABI 2016 [4].

### Related work

Among the first software tools that succeeded in counting the *k*-mers of large genome data sets was Jellyfish [5], which uses a lock-free hash table that allows parallel insertion. In the following years, several tools were published, successively reducing running time and required memory. BFCounter [6] uses bloom filters for *k*-mer counting to filter out rarely occurring *k*-mers stemming from sequencing errors. Other tools like DSK [7] and KMC [8] exploit a two-disk architecture and aim at reducing expensive IO operations. Turtle [9] replaces a standard Bloom filter by a cache-efficient counterpart. MSPKmerCounter [10] introduces the concept of minimizers to the *k*-mer counting, thus further optimizing the disk-based approach. The minimizer approach was later on refined to signatures within KMC2 [11]. Up to now, the two most efficient open source software tools that can work with small memory requirements have been KMC2 and DSK [12]. KMC2 uses a sorting based counting approach that has been optimized for *k* < 32. However, its performance drops when *k* grows larger. Instead, DSK uses a single large hash table and is therefore efficient for large *k* (but does not support *k* > 127). However, for small *k*, it is clearly slower than KMC2. A recently released *k*-mer counting tool is KCMBT [13]. By the use of multiple burst trees, KCMBT is under some conditions even faster than KMC2. However, it is restricted to *k* < 32 and its memory requirements vastly exceeds the available memory of typical desktop computers like our test systems. To the best of our knowledge, the only existing approach that uses GPUs for counting *k*-mers is the work by Suzuki et al. [14].

### Contribution

In this article we present the open source *k*-mer counting tool *Gerbil*. Our software is the result of an extensive process of algorithm engineering that tried to bring together the best ideas from the literature. The result is a *k*-mer counting tool that is both time efficient and memory frugal.^1^ In addition, *Gerbil* can optionally use GPUs to accelerate the counting step. Thus, *Gerbil* outperforms its strongest competitors both in efficiency and resource consumption.

**Fig. 1:**
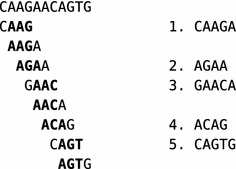
Minimizers and super-mers of the DNA string CAAGAACAGTG. Here, $k=4$ and $m=3$. For each *k*-mer, the bold part is its minimizer. The example uses the lexicographic ordering on 3-mers based on $A<C<G<T$. The sequence is divided into the five super-mers CAAGA, AGAA, GAACA, ACAG, and CAGTG that would be stored in temporary files

**Fig. 2:**
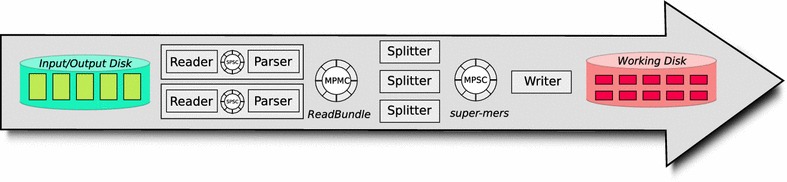
Work flow of Phase One

**Fig. 3:**
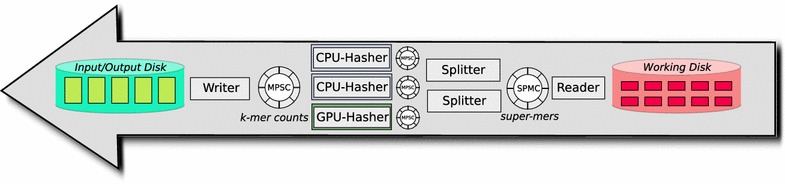
Work flow of Phase Two

**Fig. 4:**
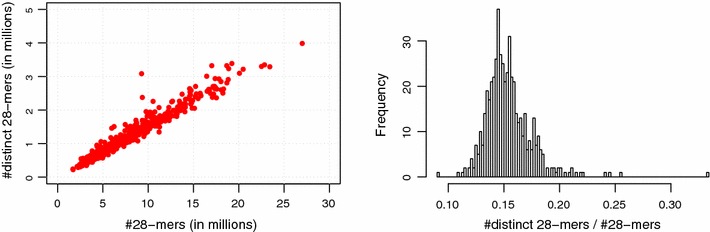
*Left* The number of *k*-mers and distinct *k*-mers of 511 temporary files that have been created while processing the *F vesca* data set for *k* = 28. A single temporary file is not shown since it contains far more *k*-mers than the other files.* Right* Distribution of the ratio between the number of distinct *k*-mers and the total number of *k*-mers for the temporary files of the *F vesca* data set and *k* = 28

**Fig. 5:**
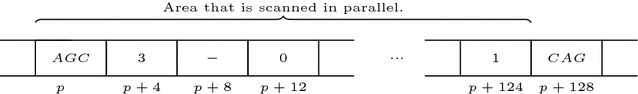
GPU memory access pattern. The* figure* shows the memory area that is being scanned while probing a hash table entry that is stored at memory address *p*. In this example, *k* = 3 and each table entry needs four bytes for the key and four bytes for the counter. Therefore, 16 entries can be loaded from global memory within one step and are scanned in parallel

**Fig. 6:**
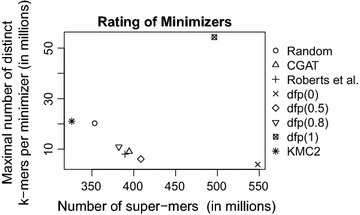
Evaluation of various total ordering strategies for minimizers (*F vesca*, *m* = 6, *k* = 28). Strategy dfp(*p*) has been tested with $p \in \{0, 0.5, 0.8, 1\}$

**Fig. 7:**
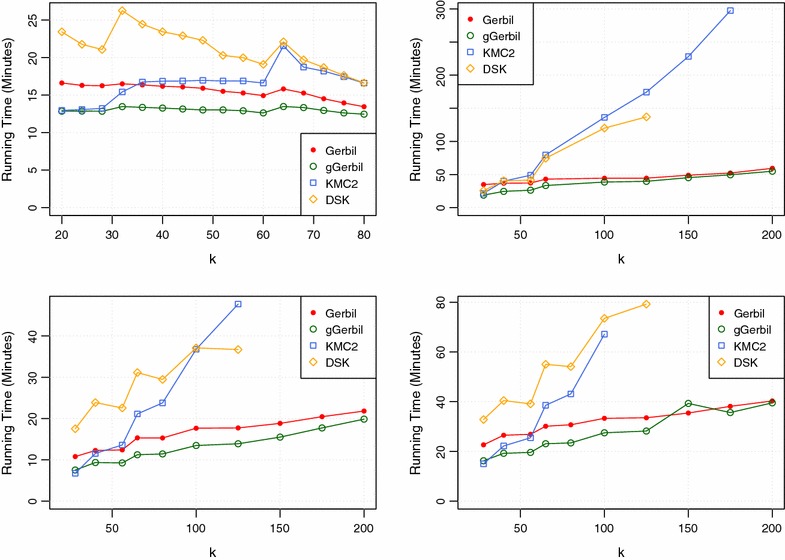
Running times on Test System Two.* Top left*: *G. gallus*,* top right*: *GRCh38*,* bottom left*: *N. crassa*,* bottom right*: *A. thaliana*

The software is written in C++ and uses CUDA for GPU programming. It is freely available at https://github.com/uni-halle/gerbil under MIT license.

### Structure

In the next section we describe the general algorithmic work flow of *Gerbil*. In the main part of this article, we focus on algorithm engineering aspects that proved essential for high performance and describe details, like the integration of a GPU into the counting process. Afterwards, we evaluate *Gerbil*’s performance in a set of experiments and compare it with those of KMC2 and DSK. We conclude this article by a short summary and a glance on future work.

## Work-flow

*Gerbil* uses a two-disk approach that is similar to those of most contemporary *k*-mer counting tools [7, 10, 11]. The first disk contains the input read data and is used to store the counted *k*-mer values. We call this disk input/output-disk. The second disk, which we call working disk, is used to store temporary files that are created and removed during runtime.

To make optimal use of the hardware, *Gerbil* is designed as a parallel program, with multiple threads running concurrently. Although the following description of the main work flow is presented sequentially, all of the steps are interleaved and therefore executed in parallel. This is done by a classical pipeline architecture. Each output of a step makes the input of the next. A couple of specialized buffers are used to connect the steps of the pipeline. Such buffers are designed for all combinations of single or multiple producers (SP/MP) and single or multiple consumers (SC/MC). The actual number of parallel threads depends on the system and is determined by the software at runtime to achieve optimal memory throughput.

**Table 1:** Test systems

	System one	System two
CPU	Intel Core-i5 2550k	Intel Xeon(R) E3-1231v3
Threads	4	8
RAM	16 GB DDR3	32 GB DDR3
GPU	GeForce GTX 970	GeForce GTX TITAN X
Working-disk	256 GB Crucial M550	2x Samsung 850 EVO 500 GB (RAID-0)
OS	Ubuntu 16.04 LTS	Ubuntu 16.04 LTS
In/out-disk	Transcend StoreJet 35T3 USB 3.0 (External HDD)	Transcend StoreJet 35T3 USB 3.0 (External HDD)

*Gerbil* is divided into two phases: distribution and counting. Next, we give a high-level description of both phases.

### Distribution

Whole genome data sets typically do not fit into the main memory. Hence, the goal of the first phase is to split the input data into a couple of smaller temporary files. The key idea is to assure that the temporary files partition the input genome data in such a way, that all occurrences of a certain *k*-mer are stored in the same temporary file. This way, one can simply count the *k*-mers of the temporary files independently of each other, with smaller main memory requirements. To split the genome data into temporary files, we make use of the *minimizer* approach that has been proposed by [15] and later on refined by [11]: a genome sequence can be decomposed into a number of overlapping super-mers. A *super-mer* of a genome sequence is defined as a substring of maximal length such that all *k*-mers on that substring share the same minimizer. Hereby, a *minimizer* of a *k*-mer is defined as a substring of fixed length *m* that is minimal with respect to some total ordering on strings of length *m*. Thus, contiguous *k*-mers of a genome read are joined to a super-mer if they share the same minimizer. By storing super-mers with the same minimizer in the same temporary file, we guarantee that all occurrences of identical *k*-mers end up in the same temporary file.

**Table 2:** Data sets (Additional file 1)

Data set	Format	Size (GB)	$\varnothing$ Read length	# 28-mers	# Distinct 28-mers	Ratio (%)
*F. vesca*	FASTQ	10.2	352.1	4,134,078,256	632,436,468	15
*M . balbisiana*	FASTQ	98.6	100.0	20,531,572,597	965,691,662	4
*G. gallus*	FASTQ	115.9	100.0	25,337,974,831	2,727,529,829	11
*H. sapiens*	FASTQ	223.3	100.0	62,739,461,708	6,336,805,684	10
*H. sapiens 2*	FASTQ	339.5	100.0	98,892,620,173	6,634,382,141	7
*GRCh38*	FASTA	100.0	1000.0	97,300,000,000	1,802,953,276	2
*N. crassa*	FASTA	23.3	7778.3	22,808,741,626	21,769,513,655	95
*A. thaliana*	FASTQ	72.7	4804.6	35,905,278,785	32,894,281,429	92

The rightmost column ’Ratio‘ describes the ratio between the number of distinct 28-mers and the total number of 28-mers

See Fig. 1 for an example.

Similar to related approaches, *Gerbil* counts the *canonical* representations of *k*-mers. Since DNA is organized in double helix form, each *k*-mer $x \in \{A,C,G,T \}^k$ corresponds to its *reverse-complement* *y* which is defined by reversing *x* and replacing $A \Leftrightarrow T$ and $C \Leftrightarrow G$. Many applications do not distinguish between a *k*-mer and its reverse-complement. Thus, *Gerbil* uses the lexicographically lesser of *x* and *y* as the canonical representation of both. This reverse-complement normalization can be turned off by command flag.

The work-flow of the first phase is described by the following steps. See Fig. 2 for a visualization.

1. A group of *reader threads* read the genome reads from the input disk into the main memory. For compressed input, these threads also decompress the input.
2. A group of *parser threads* convert the read data from the input format into an internal read bundle format.
3. A group of *splitter threads* compute the minimizers of all *k*-mers of the reads. All subsequent substrings of a read that share the same minimizer are stored as a super-mer into an output buffer.
4. A single *writer thread* distributes the super-mers to one of multiple temporary files that are stored at the working disk. Hereby, all super-mers with the same minimizer are assigned to the same temporary file.

### Counting

After the first phase has been completed, the temporary files are sequentially re-read from working disk. The counting of *k*-mers is typically done by one of two approaches: Sorting and Compressing [11] or using a hash table with *k*-mers as keys and counters as values [5, 7]. The efficiency of the sorting approach typically relies on the sorting algorithm Radix Sort, whose running time increases with the length of *k*-mers. Since we aim at high efficiency for large *k*, we decided to implement the hash table approach. Therefore, we use a specialized hash table with *k*-mers as keys and counters as values. We use a hash table that implements open addressing and solves collisions via quadratic hashing. Alg. 1 shows a high level description of the insertion method.

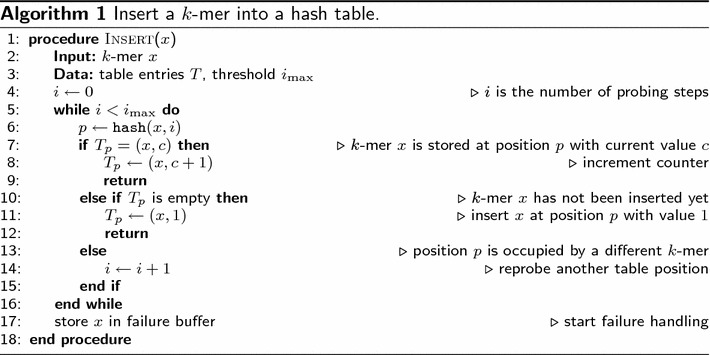



The number of probing operations during the insertion of *k*-mers in a hash table has a crucial influence on the efficiency of the whole process. Experimentally, we observed that the average number of probings is quite small (about 1.5 probings per *k*-mer in the *F vesca* data set.) However, a few *k*-mers may need a very high number of probings until a match or an empty entry is detected. Since these *k*-mers would slow down the whole process, we stop the probing of the hash table after a constant number $i_{\max }$ of trials. To prevent *k*-mers from getting lost, *Gerbil* stores such *k*-mers in failure buffers that are represented by additional temporary files at the working disk. The value $i_{\max }$ is supposed to be roughly $\log _2 n$, where *n* is the total number of *k*-mers in a data set. Thus, the whole insertion process has a running time of $\mathcal {O}(n \log n)$. Since *n* is in the order of several billions in most real-world data sets, we found that $i_{\max }=30$ works well in most cases. Figure 3 visualizes the work-flow of the second phase. The following steps are executed for each temporary file.

1. A single *reader thread* reads the super-mers from the temporary file and stores them in main memory.
2. A group of *splitter threads* split the super-mers into *k*-mers. Each *k*-mer is distributed to one of multiple hasher threads by considering the hash value of each *k*-mer. This ensures that multiple occurrences of the same *k*-mer are assigned to the same hasher thread.
3. A group of *hasher threads* insert the *k*-mers into their thread-own hash tables. Thus, every hasher thread maintains its own hash table. After a temporary file has been completely processed, each hasher thread sends the content of its hash table to an output buffer.
4. A single *writer thread* writes the final *k*-mer counts to the output disk.

## Algorithm engineering

In this section, we want to point out several details on the algorithm engineering process that were essential to gain high performance. Since the more delicate problems are located in the counting step, most of these details refer to the second phase.

### Hash functions

The efficiency of the hash table approach relies on a well-chosen hash function that maps *k*-mers to integers. *Gerbil* uses two different hash functions for different purposes. In general, the combination of two independent hash functions leads to a more uniform distribution of *k*-mers.

**Table 3:** Running times in the format mm:ss (the best performing in italics)

Data set	*k*	System one				System two			
		Gerbil	gGerbil	KMC2	DSK	Gerbil	gGerbil	KMC2	DSK
*F. vesca*	28	02:15	*01:47*	01:51	02:53	01:37	*01:21*	01:32	02:05
	40	02:23	*01:58*	02:49	04:13	01:49	*01:31*	02:12	02:52
	56	02:31	*01:59*	03:05	03:52	01:53	*01:31*	02:30	02:50
	65	03:02	*02:12*	04:23	05:23	02:05	*01:42*	03:35	03:37
*M. balbisiana*	28	14:48	*12:04*	12:24	13:50	11:37	*10:09*	10:50	11:06
	40	13:55	*12:41*	15:50	15:30	11:35	*10:30*	13:46	12:26
	56	12:40	*11:31*	15:43	14:30	10:51	*09:55*	13:36	11:44
	65	12:58	*11:38*	18:48	16:52	10:55	*09:57*	15:47	12:34
*G. gallus*	28	21:10	*15:07*	15:26	25:44	16:14	*12:50*	13:10	21:00
	40	20:30	*16:23*	19:22	31:19	16:09	*13:15*	16:49	23:48
	56	18:58	*15:32*	19:19	24:04	15:16	*12:54*	16:48	19:59
	65	20:12	*15:50*	22:27	26:04	15:48	*13:22*	19:25	21:33
*H. sapiens*	28	46:41	32:04	*31:24*	66:16	33:54	*25:18*	26:44	50:15
	40	50:14	*37:59*	44:06	102:48	34:30	*26:40*	35:59	54:21
	56	44:05	*34:21*	43:56	60:29	34:30	*26:40*	35:25	45:32
	65	43:32	*35:46*	53:31	96:31	32:01	*26:21*	42:19	47:50
*H. sapiens 2*	28	73:29	53:55	*50:09*	146:10	54:19	*39:05*	41:47	76:50
	40	77:54	*62:12*	71:27	209:12	53:31	*42:27*	57:02	83:59
	56	67:28	*57:22*	70:46	138:06	50:25	*40:18*	56:28	72:35
	65	68:50	*58:41*	87:14	156:05	50:53	*42:28*	68:10	78:13
*GRCh38*	28	62:19	50:19	*43:46*	65:20	34:52	*18:49*	21:36	25:23
	40	69:00	*61:03*	68:47	116:08	36:52	*24:32*	39:57	40:54
	56	78:44	*70:57*	80:40	111:39	37:10	*26:15*	48:59	41:13
	65	80:43	*73:27*	114:00	225:35	42:54	*33:08*	79:34	73:25
	100	82:30	*81:35*	178:04	ME	45:34	*38:43*	136:20	114:09
	125	79:55	*77:42*	226:02	ME	44:41	*40:09*	174:28	133:56
	150	83:04	*82:33*	293:02	NS	49:03	*45:23*	TL	NS
	175	86:07	*85:51*	TL	NS	53:14	*50:35*	TL	NS
	200	93:48	*90:49*	TL	NS	60:03	*56:25*	TL	NS
*N. crassa*	28	20:29	09:55	*09:49*	25:41	10:47	07:31	*06:45*	17:31
	40	22:15	*11:50*	15:45	34:31	12:15	*09:21*	11:32	23:52
	56	23:16	*12:01*	18:15	32:03	12:24	*09:14*	13:36	22:33
	65	27:13	*16:00*	27:34	44:19	15:19	*11:13*	21:06	31:07
	80	26:26	*17:16*	31:46	41:22	15:17	*11:25*	23:47	29:27
	100	28:18	*21:09*	TL	74:57	17:39	*13:28*	36:45	37:05
	125	28:56	*22:37*	TL	76:06	17:43	*13:53*	47:41	36:42
	150	30:44	*26:18*	TL	NS	18:49	*15:30*	TL	NS
	175	33:04	* 29:01*	TL	NS	20:26	*17:43*	TL	NS
	200	37:05	*34:33*	TL	NS	21:48	*19:51*	TL	NS
*A. thaliana*	28	33:53	*19:09*	19:41	43:00	22:38	16:14	*15:00*	32:48
	40	42:41	*25:24*	28:27	55:14	26:30	*19:14*	22:11	40:27
	56	43:59	*27:53*	33:48	52:06	26:52	*19:36*	25:29	39:07
	65	48:22	*34:05*	TL	114:34	30:07	*23:05*	38:33	55:01
	80	48:24	*38:32*	TL	109:43	30:42	*23:25*	43:06	54:06
	100	51:01	*42:37*	TL	141:28	33:18	*27:29*	67:09	73:30
	125	52:17	*44:49*	DS	148:44	33:31	*28:11*	TL	79:14
	150	53:47	*49:00*	TL	NS	*35:26*	39:17	TL	NS
	175	58:55	*53:37*	TL	NS	38:07	*35:37*	TL	NS
	200	66:42	*62:31*	TL	NS	40:17	*39:31*	TL	NS

Some runs were aborted after a time limit of 5 h (TL). In addition, some runs failed due to insufficient disk space (DS) or memory errors (ME). In addition, DSK simply does not support values of *k* > 127 (NS). The label ‘gGerbil’ stands for *Gerbil* with activated GPU mode. Instead, standard ‘Gerbil’ does not use any GPU

The first hash function hash(*x*, *i*) is used for the probing of the hash table (see Alg. 1). Since the hash table has a large and variable number of entries, the function needs to cover a large range of values to reach every table position. In contrast, we use a second hash function partHash for assigning *k*-mers to hasher threads. This function is designed to map *k*-mers to a small range of values with very little computational effort. Alg. 2 shows the C++ implementation of both hash functions in a slightly simplified manner. A major difference between the implementations of hash and partHash is the number of bases that is considered. While hash uses blocks of 32 bases (64 bits) in each step, the function partHash uses smaller blocks of 8 bases (16 bits). A second difference is the running time of both functions. Whereas the running time of partHash is constant, the running time of hash grows linearly with *k*.

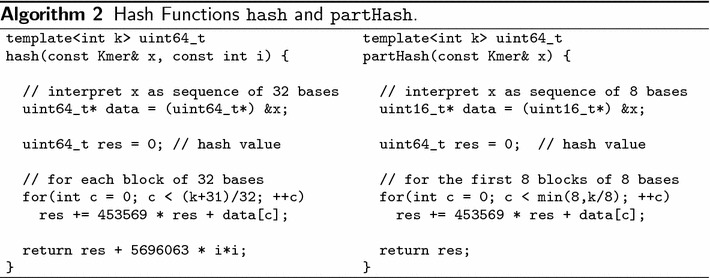



### Hash table size

A key aspect for an efficient implementation is the economic use of main memory. Therefore, we aim at estimating the expected size of each hash table as closely as possible. An economic use of main memory ensures that the operating system can use the free main memory as cache for the buffering of expensive disk operations. To find a economic size of our hash tables, we first approximate the number *d* of distinct *k*-mers in a temporary file.

#### Approximation

Since *d* is not known before processing the temporary file, we estimate this quantity using a simple linear model. In contrast to the number of distinct *k*-mers, the total number *n* of *k*-mers in a temporary file is already known from the first phase. To find an estimation for *d*, we multiply *n* with a variable $\alpha$ that describes the estimated ratio between distinct *k*-mers and the total number of *k*-mers in the current file. Since we assume that the values of $\alpha$ do not deviate much between different temporary files, we initialize $\alpha$ with the value $\alpha '$ of the temporary file that has been processed previously and adjust it at runtime. Figure  4 shows the distribution of $\alpha$ for one of our test data sets.

#### Fill level

The probing of the hash table becomes more expensive as the number of inserted elements grows. At a high fill level, most entries are occupied by non-matching *k*-mers and thus, a high number of reprobing operations is needed to insert a *k*-mer. To care for this effect, we initialize our tables to total size of $|T| = \alpha \cdot n \cdot \beta ^{-1}$ for $\beta < 1$. Thus, after the *k*-mers of a temporary file have been inserted, the tables have an expected fill level of about $\beta$. The advantage of this strategy is a small number of probing operations during the insertion process at expense of unused main memory. Experiments showed that a fill level of about $\beta =40\%$ seems to balance both aspects best.

**Table 4:** Detailed running times (in format mm:ss) and maximal main memory and disk space consumption (in GB) for the *G. gallus* instance

	*k*	System one				System two			
		Gerbil	gGerbil	KMC	DSK	Gerbil	gGerbil	KMC	DSK
Phase 1	28	10:04	10:03	10:51	10:22	09:49	09:49	09:52	09:30
Phase 2	28	10:26	06:20	04:46	16:00	06:25	03:01	03:16	11:01
Main memory	28	2.14	2.14	14.28	15.28	2.21	1.79	26.99	16.69
Disk space	28	23.66	23.66	24.86	37.30	23.66	23.66	24.86	37.30
Phase 1	56	10:01	10:06	10:40	10:26	09:49	09:49	09:47	09:30
Phase 2	56	08:56	05:25	09:08	13:13	05:27	03:05	06:59	10:00
Main memory	56	3.97	4.59	14.29	15.00	4.01	3.41	26.98	14.78
Disk space	56	16.25	16.25	17.02	57.20	16.25	16.25	17.02	57.20

Each entry is the average of three runs

#### Multiple passes

In its first phase, *Gerbil* divides the genome sequences into temporary files. Since a single temporary file is far smaller than the original genome data, it can be processed efficiently. However, it is still possible that the number of *k*-mers in a single temporary file exceeds the maximal capacity $|T_{\max }|$ of our hash tables, which is given by available main memory. In such a case, the set of *k*-mers that do not fit into the hash tables would be transfered into failure buffers and stored in additional temporary files by *Gerbil*’s failure handling. This behaviour is not optimal in cases where the size of a temporary file vastly exceeds the capacity of our hash tables. This is because in contrast to regular temporary files that contain super-mers, the additional files contain single *k*-mers. Since this would be an inefficient way to store large amounts of *k*-mers, we apply a different approach. For each temporary file, we first consider the number *n* of *k*-mers. Should this number exceed the maximal total capacity $|T_{\max }|$, we run multiple passes over the file. The total number of passes *p* is calculated by $p = \lceil n / |T_{max}| \rceil$. In each pass, *Gerbil* considers a subset of the *k*-mers by evaluating the hash function partHash on each *k*-mer. In the *i*-th pass, it considers only those *k*-mers for which $\texttt {partHash}(x) \equiv i \mod p$. Thus, after *p* passes, all *k*-mers are guaranteed to be processed.

### Load balancing

*Gerbil* has multiple hasher threads, each maintaining its own hash table. This has several advantages. One major advantage is the distribution of the hash tables to separated memory spaces like main memory and GPU memory. Here, we discuss the problem of assigning *k*-mers to the hasher threads. *Gerbil* uses the value of partHash of each *k*-mer to determine the id $t_{\mathrm{id}}$ of a hasher thread. In the most simple form, the *k*-mers could be distributed uniformly to all hasher threads by selecting $t_{\mathrm{id}}(x) = \texttt {partHash}(x) \mod N$, where *N* is the number of hasher threads. However, as shown by experiments, GPU hasher threads are often far more efficient than CPU hasher threads. A uniform distribution of *k*-mers between all hasher threads is therefore not desirable. A better approach is to assign a number of *k*-mers to each thread that is proportional to its throughput. Thus, more work is assigned to GPU hasher threads. To do so, we constantly measure the throughput of each hasher thread, i. e. the time needed to insert and extract a fixed number of *k*-mers. Whenever a new temporary file is loaded from disk, we re-balance the number of *k*-mers that are assigned to each hasher thread, considering the throughput and capacity of each hash table. By that, we automatically determine a good division of work between hasher threads without the need of careful hand-tuning.

### GPU integration

*Gerbil* can use GPUs to speed-up the counting step by enabling additional GPU hasher threads. To integrate one or more GPUs into the process of *k*-mer counting, several problems have to be dealt with. Typically, a GPU performs well only if it deals with data in a highly parallel manner. In addition, memory bound tasks (i. e. tasks that do not require a lot of arithmetic operations) like the counting of *k*-mers require a carefully chosen memory access pattern to minimize the number of accesses to the GPU’s global memory. We decided to transfer the hash table based counting approach to the GPU. When compiled and executed with GPU support, *Gerbil* automatically detects CUDA capable GPUs. For each GPU, *Gerbil* adds a GPU hasher thread which maintains its own hash table in GPU memory. Each GPU hash table is similar in function to a traditional hash table. However, there are two major differences in design.

1. Unlike the CPU hasher thread approach, a GPU hasher thread adds a large number of *k*-mers in parallel. Therefore, the insertion procedure is slightly changed. First, a bundle of several thousand *k*-mers is copied to the GPU global memory space. The set of *k*-mers is divided into bundles of nearly equal size. Then, we launch a large number of CUDA blocks, each consisting of 32 or more threads. Each CUDA block is responsible for the insertion of one *k*-mer bundle into a common GPU hash table.
  In contrast to the CPU hasher threads, the GPU hasher threads do not probe a single position of the hash table in each step. When probing a table position *p*, a GPU hasher thread additionally scans adjacent table positions in a range of 128 bytes (see Fig. 5). Due to the architecture of a GPU, this can be done within the same global memory access. Thus, we scan up to 16 table entries in parallel, thereby reducing the number of accesses to a GPU’s global memory. In addition, the total number of probing operations is drastically reduced. To eliminate race conditions between CUDA blocks, we synchronize the probing of the hash table by using atomic operations to lock and unlock hash table entries. Since such operations are efficiently implemented in hardware, a large number of CUDA blocks can be executed in parallel.
2. A second difference to CPU hasher threads is failure handling. Instead of directly evacuating failed *k*-mers into failure buffers, GPU hasher threads use free GPU memory to store *k*-mers that could not be inserted after the maximal number of trials. After all *k*-mers of a temporary file have been processed, the *k*-mers in this area are counted via sorting and compression. A problem occurs when the GPU memory is exhausted. In such cases, *Gerbil* copies the *k*-mers back to main memory and stores them in failure buffers, similarly to CPU hasher threads.

### Length of minimizers

The length *m* of minimizers is a parameter that has to be chosen with care. However, we can consider a basic rule: The larger *m* is chosen, the less likely it becomes that consecutive *k*-mers share the same minimizer. Therefore, the number of super-mers increases with growing *m*. An advantage of a large number of super-mers is that the set of super-mers can be distributed to temporary files more uniformly, which results in temporary files of approximately uniform size. However, a major drawback of a large number of super-mers is the increased total size of all temporary files. Thus, a smaller *m* results in a better data compression. In our experiments, we found that choosing minimizer length *m* = 7 is efficient for most data sets.

### Total ordering on minimizers

The choice of a total ordering has large effects on the size of temporary files and thus, also on the performance. To find a good total ordering, we have to balance various aspects. On the one hand, the total number of resulting super-mers are to be minimized to reduce the total size of disk memory that is needed by temporary files. On the other hand, the maximal number of distinct *k*-mers that share the same minimizer should not be too large since we want an approximately uniform distribution of *k*-mers to the temporary files. An “ideal” total ordering would have both a large total number of super-mers and a small maximal number of distinct *k*-mers per minimizer. Since these requirements contradict each other, we experimentally evaluated the pros and cons of various ordering strategies.

- CGAT: the lexicographic ordering of minimizers based on $C< G< A < T$.
- Roberts et al.  [16]: they propose the lexicographic ordering of minimizers with respect to $C<A<T<G$. Furthermore, within the minimizer computation all bases at even positions are to be replaced by their reverse complement. Thus, rare minimizers like *CGCGCG* are preferred.
- KMC2: the ordering that is proposed by [11] is a lexicographic ordering with $A<C<G<T$ and some built-in exceptions to eliminate the large number of minimizers that start with *AAA* or *ACA*.
- Random: a random order of all strings of fixed length *m* is unlikely to have both a small number of super-mers and a highly imbalanced distribution of distinct *k*-mers. It is simple to establish, since we do not need frequency samples or further assumptions about the distribution of minimizers.
- Distance from Pivot (dfp($\mathbf {p}$)): to explain this strategy, consider the following observations: Ascendingly sorting the minimizers by their frequency favors rare minimizers. As a consequence, the maximal number of distinct *k*-mers per minimizer is small. However, the total number of super-mers can be very large. Similarly, an descendingly sorted ordering results in quite the opposite effect. To find a compromise between both extremes, we initially sort the set of minimizers by their frequency. Since the frequencies depend on the data set, we approximate them by taking samples during runtime. We fix a pivot factor $0\le p \le 1$ and re-sort the minimizers by the absolute difference of their initial position to the pivot position $4^mp$. The result is an ordering that does neither prefer very rare nor very common minimizers and therefore makes a good compromise.

See Fig. 6. The value on the *x*-axis corresponds to the expected temporary disk memory, whereas the value on the *y*-axis is correlated with the maximal main memory consumption of our program. A perfect strategy would be located at the bottom left corner. Several strategies seem to be reasonable choices. We evaluated each strategy and found that a small number of super-mers is more important than a small maximal number of *k*-mer per minimizer for most data sets. As a result, we confirm that the total ordering that is already been used by KMC2 is a good choice for most data sets. Therefore, *Gerbil* uses the strategy from KMC2 for its ranking of minimizers.

## Results

### Experimental setup

We tested our implementation in a set of experiments. For each of our test data sets we counted the *k*-mers for a set of different *k* and compared *Gerbil*’s running time with those of KMC2 in version 2.3.0 and DSK in version 2.0.7. To judge performance on various types of hardware, we executed the experiments on two different desktop computers. See Table 1 for details about the hardware configuration of the test systems.

### Data sets

To get a fair comparison to KMC2 and DSK, we used the same set instances as Deorowicz et al. [11]. To test our tool for large *k*, we used additional genome reads with long read length [17]. In addition, we used a synthesized test set *GRCh38*, created from Genome Reference Consortium Human Reference 38, from which we uniformly sampled *k*-mers of size 1000. The purpose of these data sets is to have longer reads allowing to test the performance for larger values of *k*. Table 2 gives an overview of all test data sets.

### Running time

 Table 3 and Fig. 7 show the results of the performance evaluation. We want to point out several interesting observations.

- *Gerbil* with GPU support (*gGerbil*) is the most efficient tool in almost all cases. Exceptions occur for small *k* = 28, where the sorting based approach KMC2 is sometimes slightly more efficient.
- For data sets with small read length like *G gallus*, the running time of each tool decreases with growing *k* (see top left part of Fig. 7). In addition, one can observe the erratic increase of running time near *k* = 32 and *k* = 64 for all tools, due to a change of the internal *k*-mer representation.
- When *k* grows, KMC2 becomes more and more inefficient, while *Gerbil* stays efficient. When counting the 200-mers in the *GRCh38* data set, KMC2 did not finish within 20 h, whereas *Gerbil* finishes in about 1 h. The running time of DSK grows similarly fast as that of KMC2. Recall that DSK does not support values of *k* > 127 (see top right part Fig. 7).
- For small *k*, the use of a GPU improves the running time by a significant amount of time. However, with growing *k*, the data structure that stores *k*-mers grows larger. Therefore, the number of table entries that can be scanned in parallel decreases. Thus, the load balance will distribute less *k*-mers to a GPU. Experimentally, we found that the GPU induced speedup nearly vanishes when *k* exceeds 150.

### Memory and disk space

We gain some additional interesting insights when we take a closer look into Table 4 that shows detailed information on running time and memory usage of each tool.

- The use of a GPU accelerates *Gerbil*’s second phase by up to a factor of about two. However, since a GPU only affects the second phase, the overall speedup is moderate.
- All tools were called with an option that sets the maximal memory size to 14 GB on test system one and 30 GB on test system two. However, *Gerbil* typically uses much less memory due to its dynamic prediction of the hash table size. In contrast, both KMC2 and DSK use a significantly larger amount of main memory.
- *Gerbil*’s disk usage is comparable to KMC2’s disk usage, whereas the disk usage of DSK is much larger.
- *Gerbil*’s frugal use of disk- *and* main memory is a main reason for its high performance. The use of little main memory gives the operating system opportunity to use the remaining main memory for buffering disk operations. A small disk space consumption is essential since disk operations are far more expensive than the actual counting.

## Conclusions

We introduced the *k*-mer counting software *Gerbil* that uses a hash table based approach for the counting of *k*-mers. For large *k*, a use case that becomes important for long reads, we are able to clearly outperform the state-of-the-art open source *k*-mer counting tools, while using significantly less resources. We showed that *Gerbil*’s running time can be accelerated by the use of GPUs. However, since this only affects the second phase, the overall additional speedup is moderate. As future work, we plan to optimize the processing of compressed genome sequences. Another option for further speed-up would be to give up exactness by using bloom filters.

## Appendix

### Details on DNA sequence handling

We want to give some details on general aspects of our handling of DNA sequences.

#### Undetermined bases

DNA reads typically contain bases that could not been identified correctly during the sequencing process. Usually, such bases are marked *N* in FASTQ input files. In accordance with established *k*-mer counting tools, we ignore all *k*-mers that contain an undetermined base.

## Input formats

*Gerbil* supports the following input formats of genome read data: FASTQ, FASTA, staden, as well as compressed files of these formats. To process multiple files, it can also process simple text files that contain paths to one or more input files, with one path per line.

## Output format

*Gerbil* uses a binary output format that is easy to parse and requires little space. The counter of each occuring *k*-mer is stored in binary form, followed by the corresponding byte-encoded *k*-mer. Each four bases of a *k*-mer are encoded in one single byte. We encode A with 00, C with 01, G with 10 and T with 11. Most counters of *k*-meres are slightly smaller than the coverage of the genome data. We exploit this property by using only one byte for counters less than 255. A counter greater than or equal to 255 is encoded in five bytes. In the latter case, all bits of the first byte are set to 1. The remaining four bytes contain the counter in a conventional 32-bit unsigned integer. Examples (X is undefined bit):

- 67 AACGTG $$\Rightarrow$$ 01000011 00000110 1110XXXX
- 345 TGGATC $$\Rightarrow$$ 11111111 00000000 00000000 00000001 01011001 11101000 1101XXXX

When called with command line argument -x h, *Gerbil* additionally creates a human readable csv file that includes a histogram of the *k*-mer counts, i.e. the number of occurrences of each count. This option can be used to gain a general overview of the *k*-mer distribution of a data set.

## **Additional file**

**Additional file 1.** The additional file contains links to all test data sets used in the experiments of the paper.

## Footnotes

1 The tool is named *Gerbil* because of its modest resource requirements, which it has in common with the name-giving mammal.
